# Attitude of Iranian medical specialty trainees toward providing health care services to patients with mental disorders

**DOI:** 10.3389/fpsyt.2022.961538

**Published:** 2022-07-28

**Authors:** Sana Movahedi, Seyed Vahid Shariat, Mohammadreza Shalbafan

**Affiliations:** Mental Health Research Center, Psychosocial Health Research Institute, Department of Psychiatry, School of Medicine, Iran University of Medical Sciences, Tehran, Iran

**Keywords:** social stigma, community psychiatry, mental illness, medical students, medical education

## Abstract

**Introduction:**

The stigma of mental illness has a negative impact on the diagnosis and treatment of these disorders. Considering the high prevalence of mental illness, the attitude of medical specialists toward mental disorders, who are front liners in diagnosing and treating these patients, is critical. Therefore, we examined the attitude of Iranian medical specialty trainees toward providing health care services for patients with mental illness.

**Methods:**

We included 143 residents in the fields that have the most interactions with patients with mental disorders, including internal medicine, surgery, neurology, cardiovascular diseases, and psychiatry. A demographic checklist, as well as the opening minds scale for health care providers stigma assessment questionnaire, was provided, which measures five dimensions of improvement, social responsibility, social distance, exposure, and other (such as risk) in health care providers toward delivering the healthcare services to patients with mental disorders.

**Results:**

The mean score of stigma for mental illness in medical specialty trainees was 61.36 ± 4.83 out of 100. Psychiatric residents have the least stigmatizing attitude (58.38 ± 3.54), and internal medicine and cardiology residents have the highest score, respectively, (62.96 ± 6.05, 62.45 ± 3.80). As for comparing subscales between specialties, only the social responsibility subscale showed a significant difference, with psychiatry having less stigma toward social responsibility (12.93 ± 2.01) than cardiology (15.09 ± 1.50) trainees.

**Conclusion:**

The attitude of medical specialty trainees toward providing health care services for patients with mental illness is not uniform; internal medicine and cardiology residents have more stigmatizing attitude, while psychiatric residents have less stigmatizing attitude. It seems that not every contact could be useful in making a better attitude toward mental illness, but it needs preconditions, like a structured contact that leads to positive outcomes. Anti-stigma interventions are needed to improve the attitude of medical specialty trainees toward providing health care services to patients with mental illness.

## Introduction

Stigma is described as a trait that society considers undesirable and distinguishes the stigmatized person from other members of the community to which they belong. Stigma persists today in attitudes toward patients with some medical and mental disorders. This is more obvious than in the medical profession (1–4). Mental illness stigma is a serious problem that affects patients and those around them, as well as health care institutions and staff working with people with mental illness. The more a person with a mental illness feels stigmatized, the lower his or her self-esteem, social adjustment, and quality of life (5, 6). Stigma also affects access to care, as people may not be willing to seek help despite mental or emotional problems, as it may be seen as a weakness or a failure (7, 8).

In addition, people with a mental disorder diagnosis suffer from the effects of discrimination in health care. Not only do people with mental illness benefit less from access to primary care, but there is evidence that physicians perform fewer physical examinations, laboratory tests, prevention, and treatment interventions on this population (9–11). For instance, general practitioners may feel less comfortable having a patient with schizophrenia than a patient with depression or diabetes. They may have a pessimistic view of the effectiveness of psychiatric treatment (12). Although stigmatization of mental illness among health care professionals has been studied less than the general population, existing evidence suggests that medical practitioners also hold a range of attitudes toward individuals with a psychiatric diagnosis like those held by the public (13). In some studies, professional experience has been associated with a more favorable attitude. Therefore, physicians who interact more with patients may understand diversity, and the point of view toward the stigmatized group members is essential for strengthening the positive attitudes (14, 15).

In Iran, studies have shown that roughly 1 out of 4 people suffer from at least one mental disorder (16). As with most other countries, a significant number of patients with mental disorders feel that they are humiliated, discriminated against, and socially rejected. This affects the patients and their families, who often experience unpleasant stigma. It is also observed that there is a correlation between the number of hospitalizations, duration of the disorder, and the type of the mental illness with the stigma that patients and their families encounter (17, 18).

Due to the high prevalence of mental disorders in the community and the negative impact of stigmatizing attitude toward this group of patients in the treatment and diagnosis of these disorders, we decided to implement the following study to use the results to help the process of diagnosis and treatment of patients with mental disorder and to uncover where more anti-stigma interventions are needed. It is a fact that, for many patients, particularly in low- and middle-income countries like Iran, the primary choice is to use a university medical health center. Knowing that medical specialty trainees carry out a large volume of the workload of these centers, this study examined the attitude of these health care providers who have the most direct interaction with patients with mental illness in Iran toward providing services for these vulnerable groups.

## Methods

### Design

This cross-sectional study was conducted at the Iran University of Medical Sciences, one of the three largest public medical universities in Tehran, Iran, in 2020 and 2021. We enrolled all medical specialty trainees of specialties that frequently are in direct contact with the patients with mental disorders, including internal medicine, surgery, neurology, cardiovascular disorders, and psychiatry. Inclusion criteria were ongoing studying at one of the courses mentioned above at Iran University of Medical Sciences, consent for participation, and a lack of a previous degree in a mental health-related major like psychology.

### Data collection

An online survey package containing two questionnaires was sent to participants *via* an online link by email and/or social media. The participants were reminded for the first time within a week and a second time a month later.

### Instruments

A personal demographic information checklist, including the following (age, gender, residency program, marital status, number of shifts per month, and self-report of personal experience with mental illness, family history of psychiatric disorder, and history of violence or serious personal problems), and the stigma assessment questionnaire.

#### Opening minds scale for health care providers

Opening minds scale for health care providers (OMS-HC) is a self-report questionnaire that evaluates attitudes and behavioral intentions toward people with mental illness. The main questionnaire was first confirmed with the Cronbach’s alpha coefficient of 0.82. The full OMS-HC contains 20 items. Each item is responded as strongly agree, agree, neither agree nor disagree, disagree, strongly disagree, and scored from 1 to 5. The total score ranges from 20 (least stigmatizing) to 100 (most stigmatizing). Items 3, 8, 9, 10, 11, 15, and 19 require reverse coding. This questionnaire consists of five dimensions: social distance (Items 1, 3, 16, 17, and 19), other concepts (overshadow of detection and dangerous; Items 2 and 15), detection (Items 4, 5, 6, 7, and 10), recovery (Items 8, 9, and 14), and social responsibility (Items 11, 12, 13, 18, and 20; 19, 20).

The OMS-HC questionnaire has been translated and validated by two groups in Iran, with the Cronbach’s alpha: 0.76 and 0.87, respectively. While the translation of Kordloo et al. contains only ten items, we considered Vaghee et al.’s translation more suitable and used it in the study (21).

### Ethical considerations

The study protocol was reviewed by the Iran University of Medical Science Ethics Committee and approved with the IR license.IUMS.FMD.REC.1399.228. The participants filled out the questionnaire voluntarily and anonymously after online consent for participation.

### Data analysis

The collected data were analyzed using the statistical software analysis tool SPSS (version 16). For describing demographic information of the participants, results were expressed as mean and standard deviation (Mean ± SD) for quantitative variables and as a percentage for categorical qualitative variables. We used ANOVA for comparing the groups of trainees. The significance level was considered less than 0.05.

## Results

A total of 143 medical specialty trainees were enrolled in the study, with a completion rate of about 70%, 58.7% female, and 41.3% male. The mean age was 30.72 (SD: 4.22). The average night shift per month for residents was 7.97 (SD: 3.51) nights. Other demographic features are presented in Table 1.

**TABLE 1:** Demographic features of participants in the study.

Residential program	Number (Frequency percentage)	Age Mean (standard deviation)	Night shift in a month (Standard deviation	Gender (Frequency percentage)		Marital status (Frequency percentage)		Personal experience with mental illness (Frequency percentage)		Family history of psychiatric disorder (Frequency percentage)		History of experiencing violence or serious personal problems (Frequency percentage)	
								
				Female	Male	Married	Single	Yes	No	Yes	No	Yes	No
*Cardiology*	33 (23.1%)	29.21 (2.65)	9.45 (2.47)	17 (51.5%)	16 (48.5%)	9 (27.3%)	24 (72.7%)	10 (30.3%)	23 (69.7%)	12 (36.4%)	21 (63.6%)	10 (30.3%)	23 (69.7%)
*Psychiatric*	31 (21.7%)	32.77 (5.34)	4.52 (2.50)	24 (77.4%)	7 (22.6%)	18 (58.1%)	13 (41.9%)	18 (58.1%)	13 (41.9%)	23 (74.2%)	8 (25.8%)	18 (58.1%)	13 (41.9%)
*Internal medicine*	30 (21%)	31.37 (4.65)	7.13 (3.68)	21 (70%)	9 (30%)	16 (53.3%)	14 (46.7%)	3 (10%)	27 (90%)	12 (40%)	18 (60%)	10 (33.3%)	20 (66.7%)
*Surgery*	25 (17.5%)	30.80 (4.34)	10.36 (2.44)	5 (20%)	20 (80%)	9 (36%)	16 (64%)	6 (24%)	19 (76%)	4 (16%)	21 (84%)	12 (48%)	13 (52%)
*Neurology*	24 (16.8%)	29.25 (2.069)	8.92 (3.02)	17 (70.8%)	7 (29.2%)	12 (50%)	12 (50%)	5 (20.8%)	19 (79.2%)	10 (41.7%)	14 (58.3%)	13 (54.2%)	11 (45.8%)
*Total*	143 (100%)	30.72 (4.22)	7.97 (3.51)	84 (58.7%)	59 (41.3%)	64 (44.8%)	79 (55.2%)	42 (29.4%)	101 (70.6%)	61 (42.7%)	82 (57.3%)	63 (44.1%)	80 (55.9%)

The correlation between the total stigma score and the demographic features was analyzed. Among the demographic variables, having a personal history of mental illness was the only associated and significantly meaningful with the total stigma score (*P*-value = 0.007, *r* = 0.150).

The mean total stigma score for mental illness in the participants was 61.36 ± 4.83 out of 100, ranging from 51 to 75 (95% C.I. 60.56–62.16). The mean score for each group is presented in Table 2. When comparing the total stigma score between different specialties, the ANOVA results showed a significant difference between other groups (*P*-value = 0.002). A further *post hoc* test was done to express the differences in detail, shown in Table 2. Psychiatric trainees have the least stigmatizing score compared to internal medicine and cardiology trainees, having the most stigmatizing score, respectively.

**TABLE 2:** Data descriptive: The mean total stigma score for mental illness among different medical residents and their comparison with one another.

Specialty	Mean ± SD	Mean difference (*P*-value) neurology	Mean difference (*P*-value) cardiology	Mean difference (*P*-value) psychiatry	Mean difference (*P*-value) surgery	Mean difference (*P*-value) internal medicine
Neurology	61.50 ± 4.56	–	−0.95455 (0.995)	3.11290 (0.083)	−0.06000 (1.000)	−1.46667 (0.977)
Cardiology	62.45 ± 3.80	0.95455 (0.995)	–	4.06745* **(0.000)**	0.89455 (0.997)	−0.51212 (1.000)
Psychiatry	58.38 ± 3.54	−3.11290 (0.083)	−4.06745* **(0.000)**	–	−3.17290 (0.078)	−4.57957* **(0.008)**
Surgery	61.56 ± 4.74	0.06000 (1.000)	−0.89455 (0.997)	3.17290 (0.078)	–	−1.40667 (0.984)
Internal medicine	62.96 ± 6.05	1.46667 (0.977)	0.51212 (1.000)	4.57957* **(0.008)**	1.40667 (0.984)	–

Asterisk and bold value represented the significant meaningful data.

As for comparing different subscales of the scale within other specialties, it was only in the social responsibility subscale that there was a significant difference in different groups, with psychiatry having less stigma toward social responsibility (12.93 ± 2.01) than cardiology (15.09 ± 1.50) trainees (*P*-value < 0.001) as shown in Table 3.

**TABLE 3:** Comparison of OMS-HC subscales between different groups.

Subscales	Mean ± SD total score	Mean ± SD neurology	Mean ± SD cardioloy	Mean ± SD psychiatry	Mean ± SD surgery	Mean ± SD internal medicine	ANOVA between groups (*P*-value)
Social distance	16.38 ± 2.12	16.62 ± 1.99	16.27 ± 1.90	15.41 ± 1.89	16.68 ± 2.56	17.06 ± 2.06	0.33
Recovery	11.02 ± 1.91	10.87 ± 1.98	11.39 ± 1.47	11.41 ± 1.92	10.40 ± 2.10	10.83 ± 2.06	0.22
Social responsibility	14.46 ± 2.24	15.041 ± 2.62	15.09 ± 1.50	12.93 ± 2.01	14.64 ± 1.84	14.73 ± 2.51	**0.00**
Detection	13.56 ± 2.37	13.70 ± 2.21	13.72 ± 1.75	12.45 ± 2.20	13.60 ± 2.21	14.40 ± 3.03	0.27
Other concepts	5.93 ± 1.32	5.25 ± 0.89	5.96 ± 1.46	6.16 ± 1.06	6.24 ± 1.33	5.93 ± 1.55	0.68

The bold value represented the significant meaningful data.

## Discussion

The mean total stigma score for mental illness in the participants in our study is 61.36 ± 4.83 out of 100, ranging from 51 to 75. Previous studies using the same 20-item OMS-HC questionnaire have shown fewer total stigma scores. For instance, a 2012 study by Kassam et al. showed that the mean total score among health care providers/trainees in Canada was 57.5 (95% C.I. 57.2–57.9). Scores ranged from 41 to 96, and the standard deviation was 4.8 (22). In another study, the median score among healthcare trainees other than medical students in Italy was 27, IQR [21;30] for the 20 items version (theoretical range, 0–80; 23). Similar studies on pharmacy students and medical students in Canada had total stigma scores of 46.7 (95% C.I. 44.5–48.4) and 48.6 (95% C.I., 47.5–49.8), respectively, (24, 25).

Being part of the middle eastern community, higher stigma scores in our study could be due to various factors such as different answering styles or sociocultural backgrounds. Cross-cultural differences, such as the importance of public opinion and conventional viewpoints, and religious environment, are significant and need further studies (26, 27).

The result of our study shows that psychiatric trainees have a less stigmatizing attitude toward patients with mental disorders compared to internal medicine and cardiology trainees. However, this finding was insignificant for surgery and neurology trainees.

Some specialties, such as cardiology and internal medicine, have higher workloads and burnout (28, 29). Increased workload and burnout may be associated with a more stigmatizing attitude toward mental health. This could be one reason for lower stigmatizing attitudes in psychiatric residents.

It is believed that patients with mental disorders are more frequently visited in cardiology and internal medicine clinics than in surgery (30–32). This finding is controversial with previous theories that more contact with patients with mental illness will reduce the stigma. One explanation could be that, in fields such as internal medicine and cardiology, the mental disorder of the patients is not systematically diagnosed and treated. As a result, patients remain unwell, and the unsatisfied doctor would keep the negative attitude that patients with mental illness can never get better and cannot have a normal life. Whereas in fields such as psychiatry, with proper treatment of patients, longitudinal assessment, and routine follow-ups, achievements are vividly seen. The response to treatment leads to recovery, balanced work, life, and a social environment. Thus, these exclusive preconditions may be the reason for the less stigmatizing attitude of psychiatric trainees (14, 15).

In other words, not every contact could help make a better attitude toward mental illness, but it needs preconditions.

When comparing the different stigma subscales, our study only shows that psychiatric trainees are less stigmatized toward social responsibility than other groups. Previous studies have shown that stigma toward social responsibility negatively affects empathy (20). An integrated relationship model has been proposed: physicians with a better experience, more excellent patient-to-physician contact, and more empathy toward them feel less uneasy with patients with mental disorders; thus, they tend to reduce their social distance from them (33).

Age, gender, marital status, and the number of shifts seem to have no meaningful relationship with stigma toward mental disorders. Our findings are consistent with previous and similar studies in Iran (34). Our results indicate that medical trainees with a personal history of medical illness have a less stigmatizing attitude toward patients with mental disorders. This finding is similar to previous studies (35, 36). Overall, it seems like personal contact has a protective factor toward manifesting less stigma toward mental disorders, such as those with personal experience, or psychiatrist trainees who work with patients with mental disorders daily. This finding is supported by similar previous results (37, 38). As discussed previously, contact does not seem to be enough. Perhaps, in these categorized groups, once they see the long-term effect of treatment on people closest to them, they feel less stigmatized toward them and people with mental illness.

### Strengths and limitations

To the best of our knowledge, our study is among the very first studies in Iran on the attitude toward providing healthcare for patients with mental disorders. Iran University of Medical Sciences is one of the largest medical universities in Iran, and so the findings may be generalized to Iranian trainees. Nevertheless, the small sample size, particularly for between-group analyses, limits the interpretation of our findings. In addition, the lack of longitudinal study for observation of participants over time and using anti-stigma interventions is another limitation of our study.

### Implications for practice, research, and policies

The development of anti-stigma programs can simultaneously target the attitudes of medical specialty trainees toward mental disorders, help-seeking, and their social behaviors toward patients with a mental disorder. These strategies can target different observations in this study. Different anti-stigma strategies, such as educational workshops, showing a movie about a patient with a mental disorder, close contact with patients with mental disorders, and group free discussion, were suggested by previous studies (4).

Future studies on larger sample sizes, among other specialties and universities, other health care community members, and qualitative methods are suggested, particularly in the medical staff with the most interaction with patients with mental disorders. In addition, the efficacy and effectiveness of anti-stigma strategies in well-designed trials among this group of healthcare providers should be evaluated in future trials.

## Conclusion

The attitude of medical specialty trainees toward providing health care services for patients with mental illness is not uniform; internal medicine and cardiology residents have more stigmatizing attitude, while psychiatric residents have less stigmatizing attitude. It seems that not every contact could be useful in making a better attitude toward mental illness, but it needs preconditions, like a structured contact that leads to positive outcomes. Personal experience with mental illness also has a positive effect on the attitude. Anti-stigma interventions to improve the attitude of medical specialty trainees toward providing health care services for patients with mental illness should be considered.

## Data availability statement

The raw data supporting the conclusions of this article will be made available by the authors, without undue reservation.

## Ethics statement

The studies involving human participants were reviewed and approved by the Ethics Committee of Iran University of Medical Science. The patients/participants provided their written informed consent to participate in this study.

## Author contributions

SM, SS, and MS: conceptualization and design. SM and MS: data collection and initial draft preparation. SM and SS: data analyses. All authors editing and review.
